# Development of a Microphysiological Cartilage‐on‐Chip Platform for Dynamic Biomechanical Stimulation of Three‐Dimensional Encapsulated Chondrocytes in Agarose Hydrogels

**DOI:** 10.1002/cpz1.70079

**Published:** 2024-12-19

**Authors:** Valtteri Peitso, Zahra Sarmadian, João Henriques, Elsa Lauwers, Carlo Alberto Paggi, Ali Mobasheri

**Affiliations:** ^1^ Research Unit of Health Sciences and Technology, Faculty of Medicine University of Oulu Oulu Finland; ^2^ chrn on‐chip biotechnologies B.V. (aka chiron) Maastricht The Netherlands; ^3^ Department of Developmental BioEngineering, TechMed Centre, Organ‐on‐Chip Centre University of Twente Enschede The Netherlands; ^4^ State Research Institute Centre for Innovative Medicine Vilnius Lithuania; ^5^ Department of Joint Surgery First Affiliated Hospital of Sun Yat‐sen University Guangzhou China; ^6^ World Health Organization Collaborating Center for Public Health Aspects of Musculoskeletal Health and Aging Université de Liège Liège Belgium

**Keywords:** cartilage, chondrocyte, microphysiological, organ‐on‐a‐chip, osteoarthritis

## Abstract

Osteoarthritis (OA) is one of the most prevalent joint diseases globally, characterized by the progressive breakdown of articular cartilage, resulting in chronic pain, stiffness, and loss of joint function. Despite its significant socioeconomic impact, therapeutic options remain limited, largely due to an incomplete understanding of the molecular mechanisms driving cartilage degradation and OA pathogenesis. Recent advances in *in vitro* modeling have revolutionized joint tissue research, transitioning from simplistic two‐dimensional cell cultures to sophisticated three‐dimensional (3D) constructs that more accurately mimic the physiological microenvironment of native cartilage. Over the last decade, organ‐on‐chip technologies have emerged as transformative tools in tissue engineering, offering microphysiological platforms with precise control over biomechanical and biochemical stimuli. These platforms are providing novel insights into tissue responses and disease progression and are increasingly integrated into the early stages of drug screening and development. In this article, we present a detailed experimental protocol for constructing a cartilage‐on‐chip system capable of delivering controlled dynamic biomechanical stimulation to 3D‐encapsulated chondrocytes in an agarose hydrogel matrix. Our protocol, optimized for both bovine and human chondrocytes, begins with Basic Protocol 1, detailing the preparation and injection of cell‐laden hydrogels into the microdevice. Basic Protocol 2 describes the application of dynamic mechanical loading using a calibrated pressurized pump. Finally, Basic Protocols 3 and 4 focus on the retrieval of the hydrogel and RNA extraction for downstream molecular analyses. This platform represents a critical advancement for *in vitro* studies of cartilage biology, enabling more precise modeling of OA pathophysiology and evaluation of experimental therapeutics. © 2024 The Author(s). Current Protocols published by Wiley Periodicals LLC.

**Basic Protocol 1**: Cartilage‐on‐chip injection

**Basic Protocol 2**: Cartilage‐on‐chip actuation

**Basic Protocol 3**: Cartilage‐on‐chip agarose hydrogel removal

**Basic Protocol 4**: Preparation of cartilage‐on‐chip for RNA extraction

## INTRODUCTION

Osteoarthritis (OA) is the most prevalent form of arthritis, characterized by the progressive degeneration of articular cartilage within synovial joints (Buckwalter et al., 2005, 2006). According to the World Health Organization, OA is one of the significant contributors to years lived with disability among musculoskeletal conditions (Hunter & Bierma‐Zeinstra, 2019; World Health Organization, 2024). This condition is primarily driven by mechanical stress, inflammation, and the failure of cartilage to repair itself (Goldring & Goldring 2007; Houard et al., 2013). OA leads to pain (O'Neill & Felson, 2018), stiffness, and decreased joint function often resulting in a significant decline in quality of life (Owens & Conaghan, 2016). OA imposes a substantial burden on health and social care systems globally (Cross et al., 2014). Research efforts are increasingly focused on understanding the molecular mechanisms of cartilage degradation in OA and developing innovative approaches, such as organ‐on‐chip models, to advance our understanding of cartilage biology, screen for new drugs, and improve treatment strategies for OA (Pitsillides & Beier, 2011; Poulet & Staines, 2016).

In recent years, organ cultures and organ‐on‐chip systems have emerged as innovative tools in OA and cartilage biology research (Yeung et al., 2018), offering physiologically relevant platforms that mimic human joint function. These microfluidic devices simulate the cellular microenvironment, allowing for the precise control of mechanical and biochemical factors (Banh et al., 2022; Ingber, 2022). Specifically, cartilage‐on‐chip systems are designed to replicate the dynamic mechanical forces experienced by cartilage (Banh et al., 2022; Li et al., 2023; Paggi, Teixeira et al., 2022) allowing us to study the progression of osteoarthritis OA under near‐physiological conditions (Jeyaraman et al., 2024). These organ‐on‐chip models not only enhance drug testing efficacy but also offer insights into tissue regeneration, inflammation, and cartilage repair, contributing to advancements in personalized medicine and therapeutic interventions for joint diseases (Ong et al., 2023).

These platforms provide key insights into how various therapeutic interventions can influence cartilage integrity, opening doors to a better understanding of disease progression and treatment optimization. Researchers can now explore cartilage behavior and degeneration and develop therapies targeting OA with greater accuracy than before.

In this article, we describe how human and bovine chondrocytes are incorporated into a dynamically loadable organ‐on‐chip platform (Paggi et al., 2020; Paggi, Hendriks et al., 2022). We provide a detailed description for loading the cell‐agarose matrix incorporated into the platform using both bovine and human chondrocytes (Basic Protocol 1). We also describe in detail how to apply mechanical stimulation using a specific pressurized pump (Basic Protocol 2). Finally, additional protocols are included for downstream biochemical analyses (Basic Protocols 3 and 4).

## STRATEGIC PLANNING

**Figure 1:**
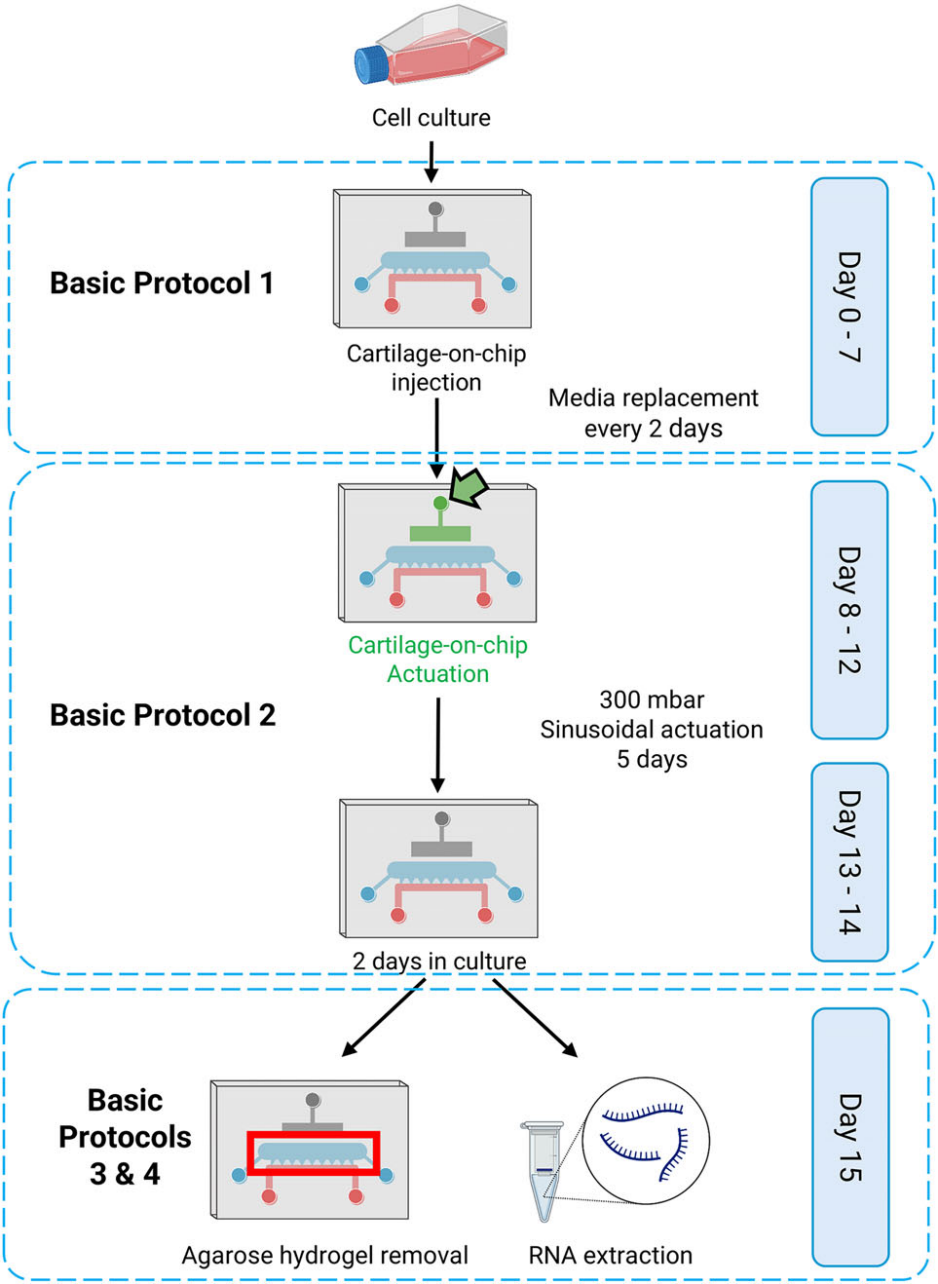
A representative overview of the workflow described in this protocol. Days 0‐7: Basic Protocol 1 involves cartilage‐on‐chip injection and maintenance with the medium being changed every 2 days. Days 8‐12: Basic Protocol 2 focuses on cartilage‐on‐chip actuation, where 300 mbar sinusoidal pressure is applied for 1 hr every day for the duration of 5 days. Days 13‐14: System in culture without actuation. Day 15: Depending on downstream analysis either Basic Protocol 3 or 4 is performed. The figure was created using BioRender.

A good indication of an average cartilage‐on‐chip injection protocol is 2 hr, but it can take up to 3 hr depending on the number of cartilage‐on‐chip devices used. Be sure to allocate sufficient time to perform all the steps without rushing. It is advisable to begin Basic Protocol 1 on a Monday to ensure that work is limited to weekdays. Figure 1 shows the representative workflow of the protocols. Refer to the Commentary section for critical parameters and time considerations.

*CAUTION*: All biological samples should be handled in a biosafety level ML‐I or ML‐II facility and under appropriate safety conditions provided by the organization or research institute.

*CAUTION*: Before placing any products into the biosafety cabinet (BSC), ensure that they are thoroughly wiped with isopropanol or ethanol. To maintain the sterility, handle all products in the BSC.

*NOTE*: All the procedures are designed for chiron's cartilage‐on‐chip devices. Any diversion or modification may compromise the success of the procedure.

*NOTE*: Incubator evaporation speeds vary. Assess the evaporation speed of your incubator before starting the experiment by adding 50, 100, 150, and 200 µl drops of medium in a Petri dish and leave it for 24 hr in the incubator.

*NOTE*: All protocols involving animals must be reviewed and approved by the appropriate Animal Care and Use Committee and must follow regulations for the care and use of laboratory animals. Appropriate informed consent is necessary for obtaining and use of human study material.

## Basic Protocol 1 CARTILAGE‐ON‐CHIP INJECTION

This protocol outlines the procedure for preparing human or bovine primary chondrocytes to be embedded in agarose hydrogel, and subsequently injected into the cartilage‐on‐chip platform. Freshly isolated chondrocytes maintain their natural phenotype, making them ideal for direct injection into the cartilage‐on‐chip platform. In contrast, chondrocytes cultured and passaged in a monolayer on a flat surface lose their natural phenotype. However, since access to freshly isolated bovine or human chondrocytes is often limited, passaged chondrocytes can be used as an alternative.

### Materials

70% (v/v) isopropanol (VWR, cat. no. 93002.1016 or equivalent)
96% (v/v) ethanol, diluted to 70% (VWR, cat. no. 20824.365 or equivalent)
Phosphate‐buffered saline (PBS) without calcium and magnesium (Thermo Fisher Scientific, cat. no. 15343571 or equivalent)
UltraPure low melting point agarose (Thermo Fisher Scientific, cat. no. 16520050 or equivalent)
Complete medium (see recipe)
0.25% trypsin‐EDTA, 1× (Gibco, cat. no. 25200‐072 or equivalent)
Human primary chondrocytes, preferably freshly isolated
Bovine primary chondrocytes, preferably freshly isolated
0.4% trypan blue solution (Sigma‐Aldrich, cat. no. 93595 or equivalent)

Water bath (VWR or equivalent)
Biological safety cabinet (BSC), i.e., laminar flow hood
Eppendorf heating block (Eppendorf or equivalent)
20‐, 200‐, and 1000‐µl calibrated micropipettes and aerosol barrier pipette tips
5‐, 10‐, and 25‐ml serological pipettes
500‐ml glass beaker, sterile
Pipetboy pipette controller
Aluminum foil
Balance
15‐ and 50‐ml conical tubes
Microwave
Cell culture flask T75, T175, or equivalent (Corning, cat. nos. 430720U, 431079 or equivalent)
Aspirator
Cell culture incubator, 37°C and 5% CO_2_
Transmitted cell culture microscope
Centrifuge
Cell counting slides (Logos Biosystems, cat. no. L12001 or equivalent)
Cell‐counting instrument (Logos Biosystems, cat. no. L40002 or equivalent)
Cartilage‐on‐chip devices (chiron, cat. no. CoC‐0001)
Hot plate (VWR or equivalent)
35‐mm Petri dish (Sigma‐Aldrich, cat. no. Z740356 or equivalent)
Eppendorf heating block
1.5‐ml microcentrifuge tubes
Cartila‐plate (chiron, cat. no. CoC‐0004)
Cartila‐plate lid (chiron, cat. no. CoC‐0002)

*CAUTION*: During agarose preparation, the phosphate‐buffered saline (PBS) and agarose solution will be warm, and the water bath will be at 57°C. Therefore, exercise caution as materials can be hot; do not touch the glass beaker without wearing proper personal protective equipment.

*NOTE*: For a successful injection, the lab room should have temperatures above 20°C. Lower temperatures will rapidly reduce the temperature of the agarose leading to premature gelation.

*NOTE*: This procedure is only meant for low melting temperature agarose, bovine, and human chondrocytes. Any diversion or modification of the procedure cannot guarantee success.

*NOTE*: Due to the varying evaporation speeds of the incubators, cartilage‐on‐chip devices may need to be cultured inside a plate filled with PBS, with the plate wrapped in parafilm (Sigma‐Aldrich, cat. no. P7793 or equivalent).

### Agarose preparation

1 Set the water bath to 57°C.
2 Clean the laminar flow hood using isopropanol or ethanol and wipe the surface area.
3 Place the Eppendorf heating block in the clean BSC and set the temperature to 37°C.
  This is a preparative step for the cartilage‐on‐chip injection.
4 Wipe down pipettes, 500‐ml glass beaker, pipette boy, and agarose container with isopropanol or ethanol, and place them in the BSC.
5 Pour 55 ml sterile PBS into the glass beaker and cover with aluminum foil.
  Use 55 ml sterile PBS instead of 50 ml, since ∼5 ml will evaporate due to boiling in the microwave.
6 Weigh an empty 50‐ml conical tube and record the measurement for later reference.
7 Place the 50‐ml tube under the laminar flow and add low melting temperature agarose powder until it reaches the 5 ml reference marking on the 50‐ml tube.
8 Weigh the 50‐ml conical tube again to confirm that the tube contains exactly 2 g agarose powder. Adjust the amount as necessary.
9 Pour the 2 g agarose powder from the 50‐ml conical tube to the glass beaker containing 55 ml PBS.
  This results in a 4% (w/v) concentration of agarose in PBS.
10 Stir the PBS‐agarose mixture using a 5‐ml serological pipette until all the large clumps are dissolved. Cover the beaker once again with aluminum foil.
11 Place the beaker in the microwave and remove the aluminum foil.
  Remember to remove the aluminum foil to prevent sparks in the microwave!
12 Turn on the microwave, use the highest power setting, and heat the mixture for 20 s.
13 Open the microwave, grab the beaker, and gently stir its contents by slowly whirling the beaker.
  Wear gloves intended for hot items when handling the glass beaker as it may be hot to touch.
14 Microwave the mixture for an additional 10 s.
15 Repeat steps 13 and 14. Afterwards, continue the microwave process in 5‐s intervals until all the clumps are dissolved.
16 When no clumps are visible in the solution, place the aluminum foil–covered beaker in the preheated water bath set to 57°C.
17 After 10 min in the water bath, the agarose should be fully dissolved and ready to use.
  The agarose must be uniformly dissolved in PBS before continuing.
18 Pipette 10 ml of agarose‐PBS mixture into a 50‐ml tube using a 10‐ml serological pipette, and place it into the water bath at 57°C.
  The agarose can be kept in the water bath for up to 4 hr after preparation.

### Cell preparation

**Figure 2:**
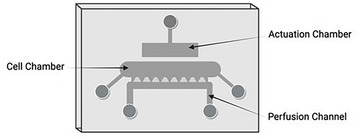
Illustrative figure of the cartilage‐on‐chip device containing the actuation chamber, cell chamber, and perfusion channel. The figure was created using BioRender.

19 Warm the complete medium and trypsin in the water bath at 37°C for 5 to 10 min. Afterward, clean the complete medium and trypsin containers with isopropanol or ethanol and place them in the BSC.
20 Retrieve the T175 cell culture flask containing human or bovine primary chondrocytes with a maximum confluency of 80% from the incubator and place it in the BSC, wipe with isopropanol or ethanol.
  Smaller volume cell culture flasks, e.g., T75 cell culture flasks, can also be used. A total of 3 × 10^6^ cells/ml is needed.
21 Aspirate the medium.
  Avoid touching the part of the flask containing the cells while aspirating the medium.
22 Pipette 10 ml PBS using a 10‐ml serological pipette. Swirl the flask gently and aspirate the PBS.
23 Pipette 5 ml trypsin using a 5‐ml serological pipette.
24 Place the flask in the incubator for 5 min.
25 Check under the microscope if the cells are detached from the surface. If not, gently tap the side of the flask and recheck under the microscope. If detachment has not occurred, return the flask to the incubator for another 2 min.
  Washing the cells with PBS without calcium and magnesium ions helps to remove trypsin inhibitors and enhance the activity of trypsin by removing calcium and magnesium ions.
26 Once the cells are detached, pipette 5 ml complete medium to the flask using a 10‐ml serological pipette.
27 Withdraw the entire volume of the flask and transfer it into a 15‐ml tube.
28 Centrifuge the 15‐ml tube containing the cells 5 min at 500 × *g*, room temperature.
29 Check for a pellet formation at the bottom of the 15‐ml tube. If no pellet is present, repeat step 28. Once the pellet has formed, place the tube in the BSC and clean it with isopropanol or ethanol.
30 Resuspend the cells in the complete medium.
31 Count the cells using a graticule or counting instrument, slides, and trypan blue. Record the count.
32 Centrifuge the 15‐ml tube containing the cells 5 min at 500 × *g*, room temperature.
33 Check for a pellet formation at the bottom of the 15‐ml tube. If no pellet is present, repeat step 32. Once the pellet has formed, place the tube in the BSC and clean it with isopropanol or ethanol.
34 Resuspend the cell pellet first with the complete medium using a 1000‐µl pipette. Pipette up and down slowly to break up the pellet and ensure the pellet is properly resuspended in the medium. Afterwards, resuspend to a final concentration of 3 × 10^6^ cells/ml using a 5‐ml serological pipette.
35 The cells are now ready for injection into the cartilage‐on‐chip device (Fig. 2).

### Cartilage‐on‐chip injection

**Figure 3:**
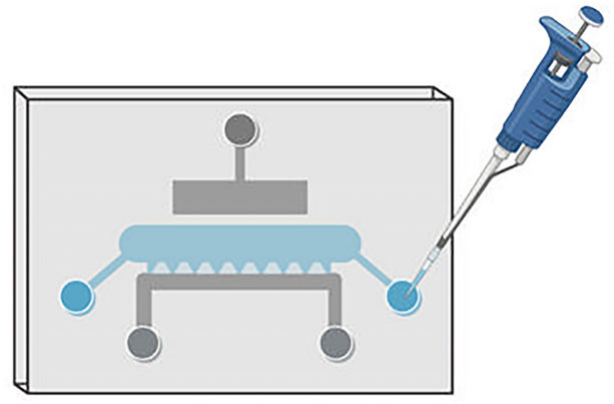
Illustrative figure of the injection of the cell‐agarose‐suspension into the cell chamber of the cartilage‐on‐chip device. The figure was created using BioRender.

36 Place the hotplate in the BSC and set it at 26°C.
  The hotplate prevents the agarose hydrogel from shrinking by allowing the agarose to cool down and solidify gradually.
37 Place the required cartilage‐on‐chip devices in the BSC and unwrap them from their package.
  Inspect devices one by one for manufacturing errors.
38 Place the cartilage‐on‐chip devices on the 26°C preheated hotplate and cover them with a Petri dish.
39 Retrieve a 50‐ml tube containing 10 ml agarose from the water bath, clean it with isopropanol or ethanol, and place it in the BSC.
40 Withdraw 400 µl agarose from the 50‐ml tube using a 1000 µl pipette, and transfer it into a 1.5‐ml microcentrifuge tube in the Eppendorf heating block set at 37°C.
  The PBS‐agarose mixture is viscous. Once the agarose is withdrawn from the 50‐ml tube, wait ≥5 s before removing the pipette tip from the mixture.
41 Repeat step 40 for three additional 1.5‐ml microcentrifuge tubes.
42 Wait 10 min for the agarose to reach 37°C.
  The agarose must be cooled to 37°C before mixing the cell suspension with agarose.
43 Withdraw 400 µl of the cell suspension using a 1000 µl micropipette into one 1.5‐ml microcentrifuge tube containing 400 µl agarose. Keep the 1.5‐ml microcentrifuge tube in the Eppendorf heating block.
  The addition of cell suspension to agarose results in a concentration of 2% (w/v) cell‐agarose suspension and 1.5 × 10^6^ cells/ml.
44 Pipette the cell‐agarose suspension up and down gently and stir the solutions using the tip of the micropipette until the two solutions are completely mixed.
45 Withdraw 50 µl cell‐agarose suspension from the 1.5‐ml microcentrifuge tube, using a 200 µl pipette. Keep the 1.5‐ml microcentrifuge tube in the Eppendorf heating block.
  This volume is sufficient to load a maximum of two cartilage‐on‐chip devices. If the cell‐agarose suspension solidifies in the tip before injection, discard the tip.
46 Gently inject ∼15 µl of the cell‐agarose suspension into the cell chamber (Fig. 3).
  Remember to put the cartilage‐on‐chip devices on the heating plate and remove the Petri dish. Do not apply too much pressure when injecting the chips; the cell‐agarose suspension can go to the perfusion channel when the mixture is injected too quickly using too much pressure.

**Figure 4:**
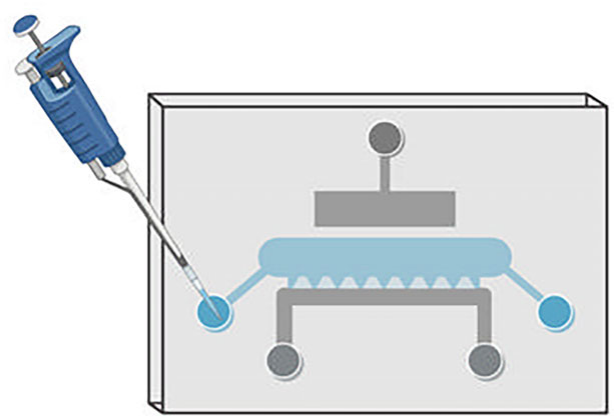
Illustrative figure of the second injection of the cell‐agarose‐suspension into the cell chamber of the cartilage‐on‐chip device. The figure was created using BioRender.

47 Repeat steps 45 and 46 for two cartilage‐on‐chip devices.
48 Inspect the devices for issues, e.g., leakage or incomplete filling of chambers.
  This step is important to verify that the injection was successful.
49 If the cell chamber is not filled, repeat steps 45 and 46, but inject from the other side of the chip (Fig. 4).

**Figure 5:**
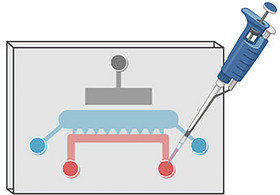
Illustrative figure of the injection of the complete medium into the perfusion channel of the cartilage‐on‐chip device. The figure was created using BioRender.

50 Once the two cartilage‐on‐chip devices have been injected with cell‐agarose suspension. Immediately discard cartilage‐on‐chip devices where the cell‐agarose suspension has entered the perfusion channel.
51 Inject 50 µl complete medium to the perfusion channel of each cartilage‐on‐chip device (Fig. 5). You must inject medium within 30 to 60 s after the cell‐agarose injection.
  Do not add too much pressure when injecting medium, since it can break the agarose membrane. The medium volume (50 µl) is intentionally larger than the channel's capacity to prevent dehydration during incubation. The additional medium should sit on top of the perfusion channel.

52 Continue injecting additional devices by repeating steps 44 to 51 until 40 to 50 µl of cell‐agarose suspension remains in the 1.5 ml‐microcentrifuge tube.
53 If additional cartilage‐on‐chip devices need to be injected, repeat steps 43 to 52.
54 Place the injected cartilage‐on‐chip devices into a Cartila‐plate and the Cartila‐plate lid.
55 Incubate the Cartila‐plate in the incubator at 37°C and 5% CO_2_.
  The cartilage‐on‐chip devices should be cultured in the incubator for 7 days, changing the medium every 48 to 72 hr before proceeding to Basic Protocol 2.

## Basic Protocol 2 CARTILAGE‐ON‐CHIP ACTUATION

**Figure 6:**
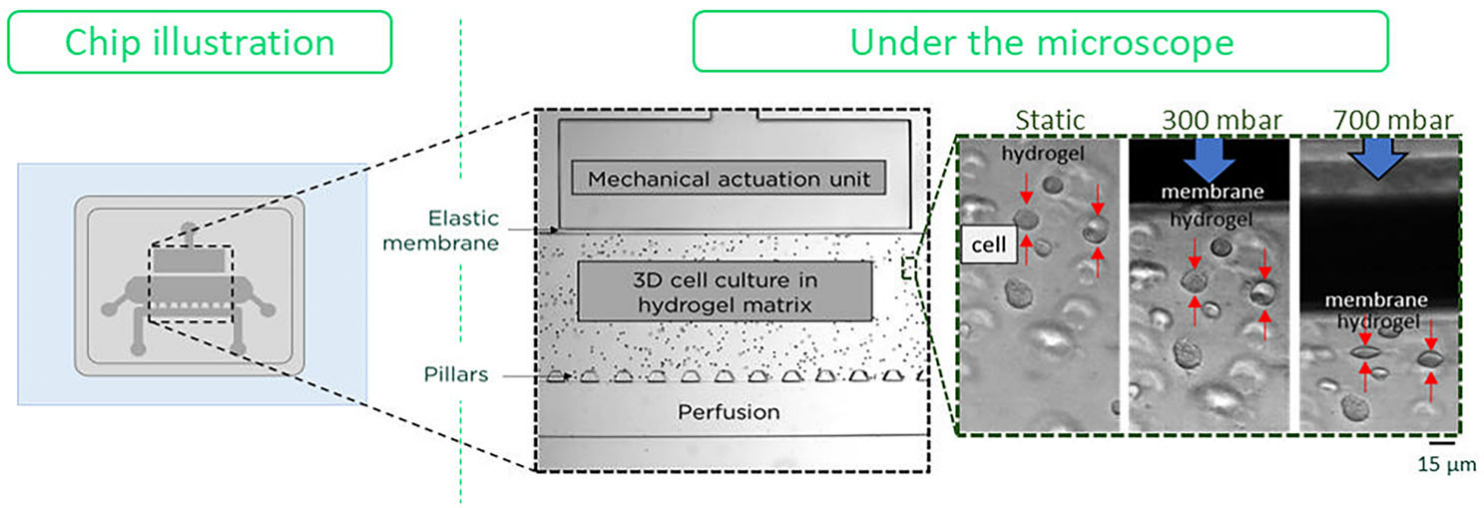
Chip illustrations under the microscope. Left: Schematic view of the chip and the area seen under the microscope. Middle: The whole view of the chip under the microscope shows the mechanical actuation unit, cell‐agarose‐hydrogel, and perfusion channel. Right: Close‐up view of cells embedded in agarose hydrogel under different conditions: Static, 300 mbar, and 700 mbar pressure (cells shown by red arrows; scale bar = 15 µm).

This protocol describes how to apply mechanical actuation stimuli to the chiron's cartilage‐on‐chip devices and connect them to a pressure controller instrument. It also includes instructions for using the pressure controller instrument. Figure 6 illustrates the cartilage‐on‐chip device under the microscope and the effect of different pressures on cell morphology. This protocol recommends using 1 Hz frequency with 300 mbar mechanical actuation, which roughly models the walking pace (Paggi, Hendriks et al., 2022).

The pressure controller instrument can be positioned inside or outside of the biological safety cabinet (BSC). The instrument can also be kept near the incubator so that the mechanical actuation can be done at 37°C. This protocol describes the actuation of 6 cartilage‐on‐chip devices in room temperature outside of BSC. However, it is recommended to perform the actuation at 37°C in the incubator.

While a 1‐hr actuation is used as a standard setting, researchers can choose different settings. However, it should be noted that using alternative settings may not guarantee the same behavior of the agarose hydrogel.

### Materials

70% (v/v) isopropanol (VWR, cat. no. 93002.1016 or equivalent)
96% (v/v) ethanol, diluted to 70% (VWR, cat. no. 20824.365 or equivalent)
Complete medium (see recipe)

Pressure controller instrument (chiron, cat. no. CoC‐0005)
Cartila‐plate (chiron cat. no. CoC‐0004)
Cartilage‐on‐chip devices (chiron cat. no. CoC‐0001)
Biological safety cabinet (BSC)
Water bath
Cartila‐plate lid (chiron cat. no. CoC‐0002)
Cartila‐plate lid with holes (chiron cat. no. CoC‐0003)
Tubing with metal wire and connector (chiron, cat. no. CoC‐0006)
Black connectors (chiron, cat. no. CoC‐0007)
Timer
Transmitted cell culture microscope
Manifold
20‐, 200‐, and 1000‐µl calibrated micropipettes and aerosol barrier pipette tips
Cell culture incubator, 37°C and 5% CO_2_

### Turning on the instrument and checking pressure levels

**Figure 7:**
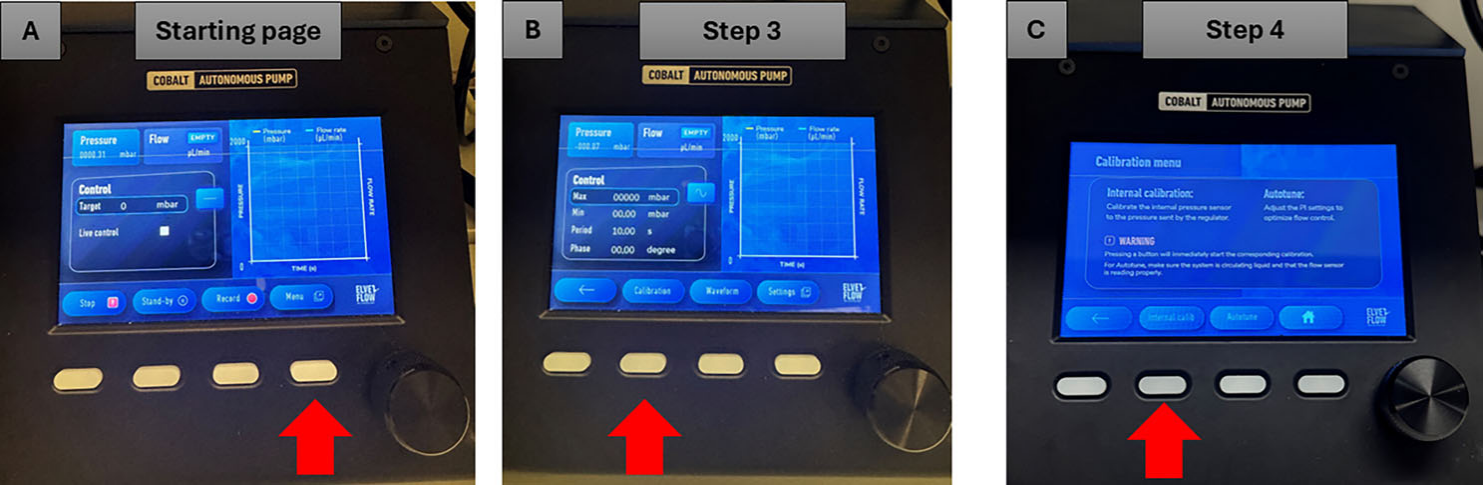
Calibration of the pressure controller instrument. Red arrows indicate the buttons to be pressed.

1 Turn on the pressure controller instrument by pressing the black power button.
  Note that the system has an integrated pressure generator.
2 Press the fourth button below the word “Menu” on the start screen page (Fig. 7A).

**Figure 8:**
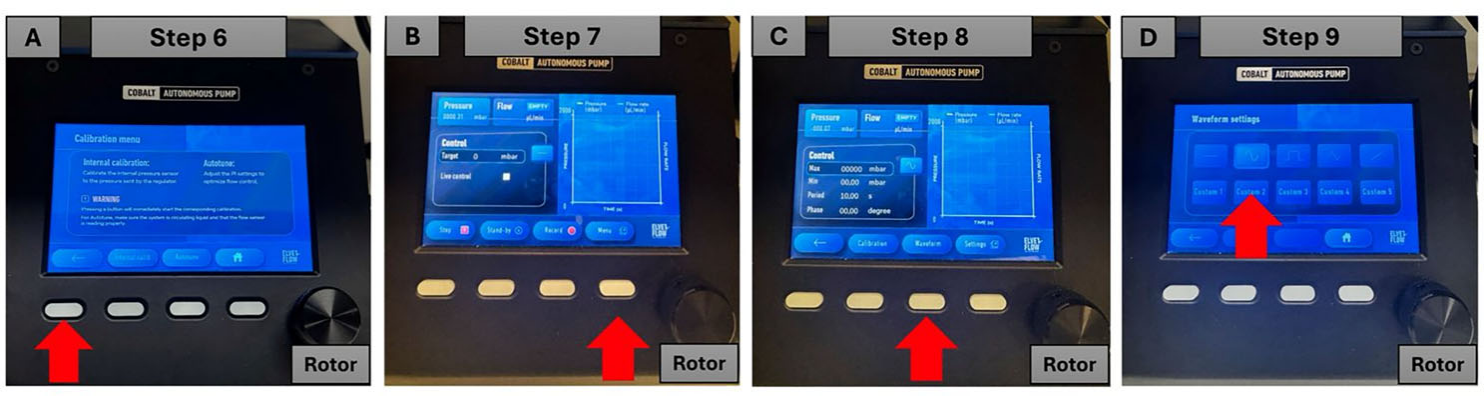
Selection of the correct waveform in the pressure controller instrument. Red arrows indicate the buttons to be pressed.

3 Press the second button below the word “Calibration” on the start screen page (Fig. 7B).
4 Press the second button below the words “Internal calib” (internal calibration) to launch the calibration process (Fig. 7C).
5 Wait the recommended 5 min for the calibration process to complete.
6 Press the first button below the red arrow symbol twice (Fig. 8A).

**Figure 9:**
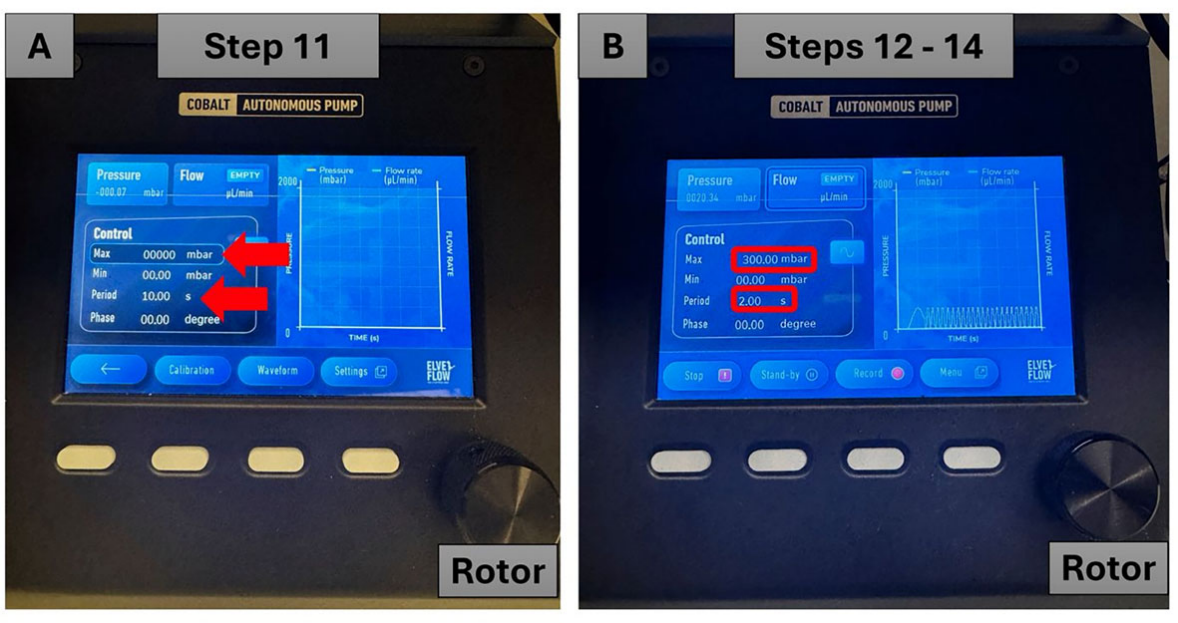
Setting the pressure and the cyclic period in the pressure controller instrument. Red arrows indicate the buttons to be pressed.

7 Press the fourth button below the word “Menu” (Fig. 8B).
8 Press the third button below the word “Waveform” (Fig. 8C).
9 Using the rotor control, choose the second option and press the rotor to select the sinusoidal waveform option (Fig. 8D).
10 Select the desired pressure and actuation levels. The default program settings in the pressure controller instrument are as follows:
  Max, 00.00 mbar;
  Min, 00.00 mbar;
  Period, 10.00 s;
  Duty cycle, 00.00.
11 Use the rotor dial to scroll up and down the screen from “Max” to “Phase” or vice versa (Fig. 9A).

12 Select “Max”, press the rotor, and adjust the pressure to 300 mbar (Fig. 9B).
13 Scroll to “Period” and press the rotor (Fig. 9B).
14 Adjust the period to 2.0 s (Fig. 9B).
  Period set to 2.0 s results in 1 Hz frequency with 300 mbar mechanical actuation.
15 The system should now be applying cyclic pressure and displaying the suggested program settings in the pressure controller instrument, as follows:
  Max, 300.00 mbar;
  Min, 00.00 mbar;
  Period, 2.00 s;
  Duty cycle, 00.00.
16 The instrument is now ready to use.
17 To stop the instrument, press the left‐most button under “Stop.”

### Connecting the cartilage‐on‐chip to the actuation unit

**Figure 10:**
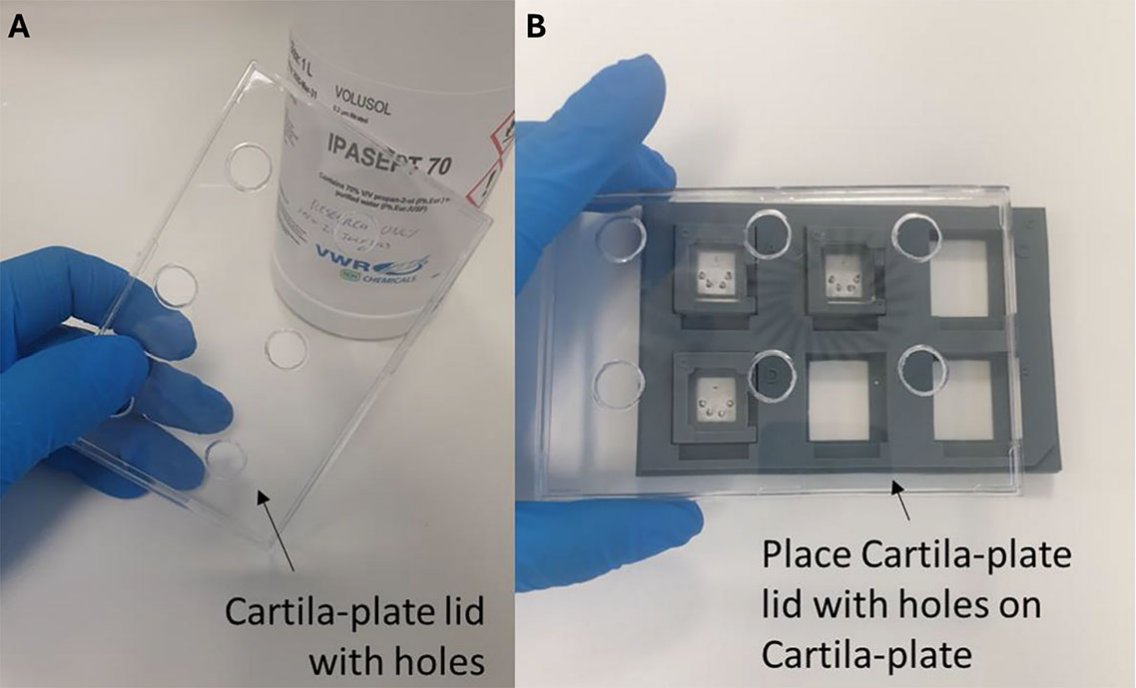
Replacing Cartila‐plate lid with Cartila‐plate lid with holes on Cartila‐plate.

18 Remove the Cartila‐plate, containing the cartilage‐on‐chip devices (from Basic Protocol 1), from the incubator. Clean the Cartila‐plate with isopropanol or ethanol before placing it in the BSC.
19 Warm the complete medium in a 37°C water bath for 5 to 10 min.
20 Remove the Cartila‐plate lid and check the amount of medium on top of the perfusion channel. If the amount is low, add 50 µl complete medium before beginning the actuation process.
  There should always be medium in the perfusion chamber and on top of the perfusion channel during the actuation, otherwise, the agarose hydrogel will dry during the process.
21 Clean the tubing with the metal wire and connector, as well as Cartila‐plate lid with holes (Fig. 10) with isopropanol or ethanol and place them in the BSC.

**Figure 11:**
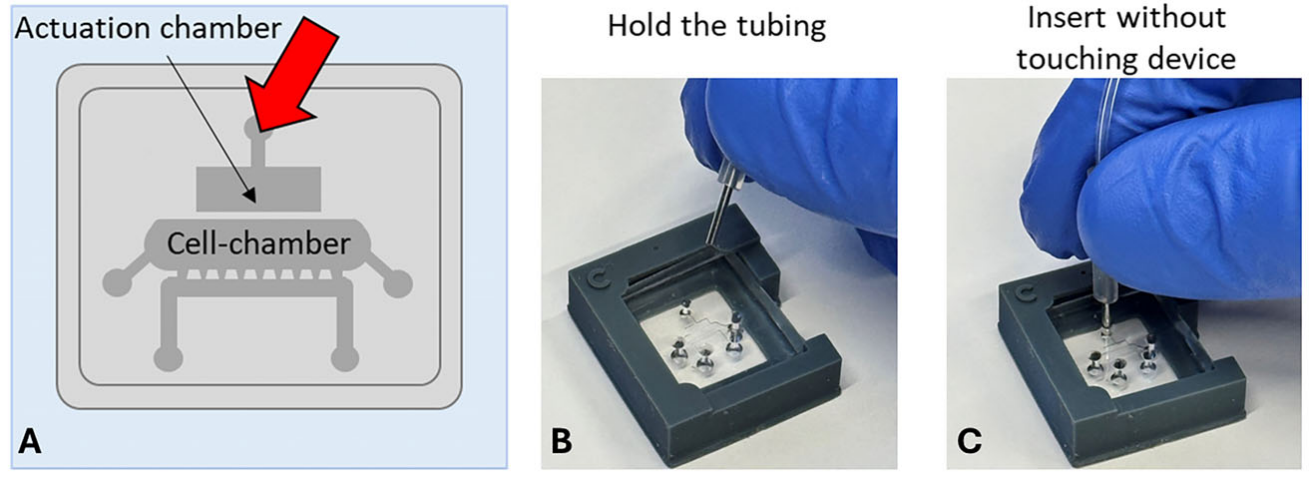
Insertion of the tubing into the hole of the actuation chamber of the cartilage‐on‐chip device. (**A**) An illustrative figure of the actuation chamber. (**B**‐**C**) Insertion of the tubing into the actuation channel.

**Figure 12:**
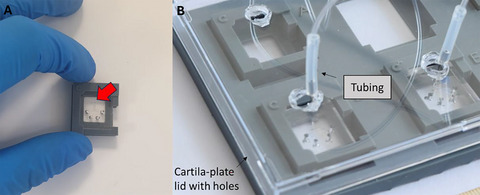
Representation of the (**A**) actuation chamber and (**B**) Cartila‐plate with cartilage‐on‐chip devices, tubing, and Cartila‐plate lid with holes.

22 Replace the Cartila‐plate lid with the Cartila‐plate lid with holes (Fig. 10). Hold the tubing close to the metal wire, thread the metal wire through the hole in the top side of the Cartila‐plate lid with holes, and then insert the tubing into the hole of the actuation chamber (Fig. 11 and Fig. 12).
  Do not apply excessive pressure with your fingers or metal wiring on the cartilage‐on‐chip material, as this will damage the agarose hydrogel inside the device. Any damage to the agarose hydrogel will result in the device being discarded.

**Figure 13:**
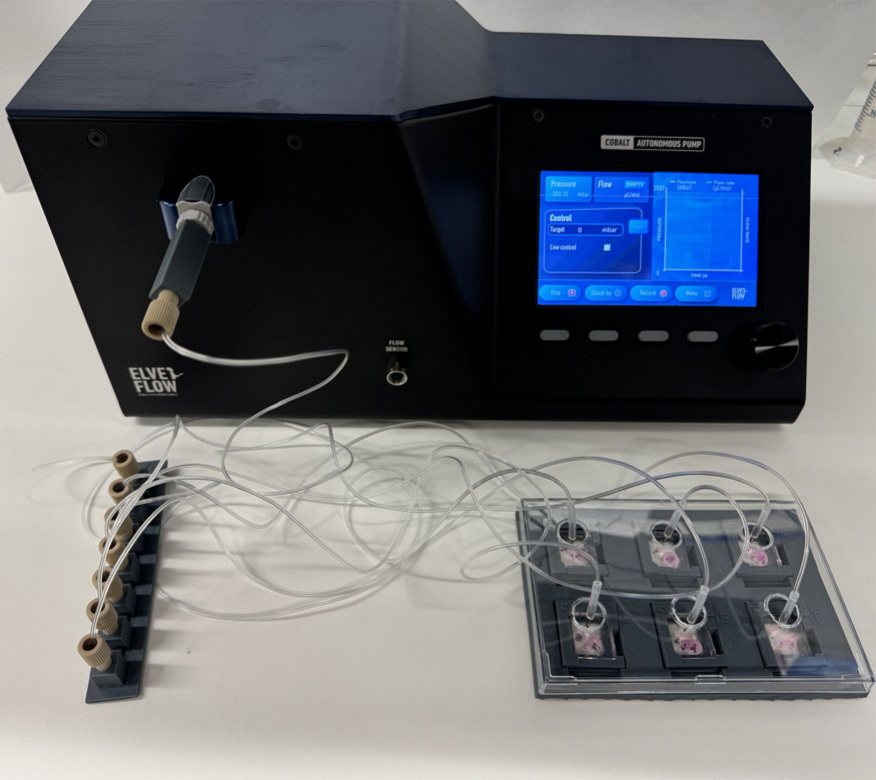
The actuation system containing a pressure controller instrument, 7‐port connector, Cartila‐plate with cartilage‐on‐chip devices, Cartila‐plate lid with holes, tubing, and connectors.

23 Repeat step 22, until 6 cartilage‐on‐chip devices are connected to the tubing.
  Up to 6 cartilage‐on‐chip devices can be connected to the pressure controller instrument.
24 Position the Cartila‐plate, with the cartilage‐on‐chip devices inside, close to the setup and screw the connector into the manifold.
25 Screw the second connector, on the other end of the tubing, to the pressure controller instrument (Fig. 13).
  Do not apply excessive force when connecting the connector to the pressure controller instrument.

26 Press the “Play” button on the pressure controller instrument and start the timer for 1 hr.
27 Inspect the proper actuation of the agarose hydrogel under the microscope.

### Unplugging the cartilage‐on‐chip from the actuation unit

28 Unscrew the connectors from the manifold, clean the Cartila‐plate with isopropanol or ethanol, and place it into the BSC.
29 Unplug the tubing with the metal wire and connector from the Cartila‐plate lid with holes.
30 Withdraw the remaining medium on top of the cartilage‐on‐chip using a 200 µl micropipette.
  Do not aspirate the medium in the perfusion channel, as doing so will damage the agarose hydrogel leading to the failure of the system.
31 Withdraw the remaining medium in the perfusion channel (between 2 and 4 µl) using a 20 µl micropipette.
32 Pipette 50 µl complete medium into the perfusion channel of the cartilage‐on‐chip using a 200 µl micropipette.
33 Repeat steps 30 to 33 for each cartilage‐on‐chip device.
34 Close the Cartila‐plate with the Cartila‐plate lid and place it in the incubator (set at 37°C and 5% CO_2_).
  The actuation is performed for 5 consecutive days, followed by a 2‐day rest period in an incubator. Static control cartilage‐on‐chip devices should be prepared and compared with the devices that have been under mechanical stimulus.

## Basic Protocol 3 CARTILAGE‐ON‐CHIP AGAROSE HYDROGEL REMOVAL

This protocol describes the procedure for removing agarose hydrogels from chiron's cartilage‐on‐chip devices. This procedure can be executed to fix agarose hydrogels after the Basic Protocols 1 and 2, enabling further specific analyses, such as immunofluorescence and immunohistochemistry. Given the delicate nature of the procedure, it is recommended that 5 to 10 test trials should be performed before starting the actual process.

### Materials

70% (v/v) isopropanol (VWR, cat. no. 93002.1016 or equivalent)
96% (v/v) ethanol, diluted to 70% (VWR, cat. no. 20824.365 or equivalent)
Phosphate‐buffered saline (PBS) without calcium and magnesium (Thermo Fisher Scientific, cat. no. 15343571 or equivalent)
4% formaldehyde (Sigma‐Aldrich, cat. no. 1004965000 or equivalent)

Biological safety cabinet (BSC)
Cartila‐plate (chiron, cat. no. CoC‐0004)
Cartila‐plate lid (chiron, cat. no. CoC‐0002)
Cartilage‐on‐chip device (chiron, cat. no. CoC‐0001)
Scalpel blade, no. 10 (Andwin Scientific cat. no. EF7281 or equivalent)
Razor blade
Forceps curved end (160‐mm size recommended but any size can be used)
Glass slide (Thermo Fisher Scientific, cat. no. 22‐042‐941 or equivalent)
20‐, 200‐, and 1000‐µl calibrated micropipettes and aerosol barrier pipette tips
Tissue wipe

*CAUTION*: During the removal of the agarose hydrogel, a sharp scalpel blade is used to separate the two parts of the cartilage‐on‐chip device. Therefore, caution is required when handling the blade.

*NOTE*: It is important to note that this protocol has not been verified to be used with other hydrogels.

*NOTE*: This protocol is exclusive for the removal of agarose hydrogels from chiron's cartilage‐on‐chip devices.

### Agarose removal

**Figure 14:**
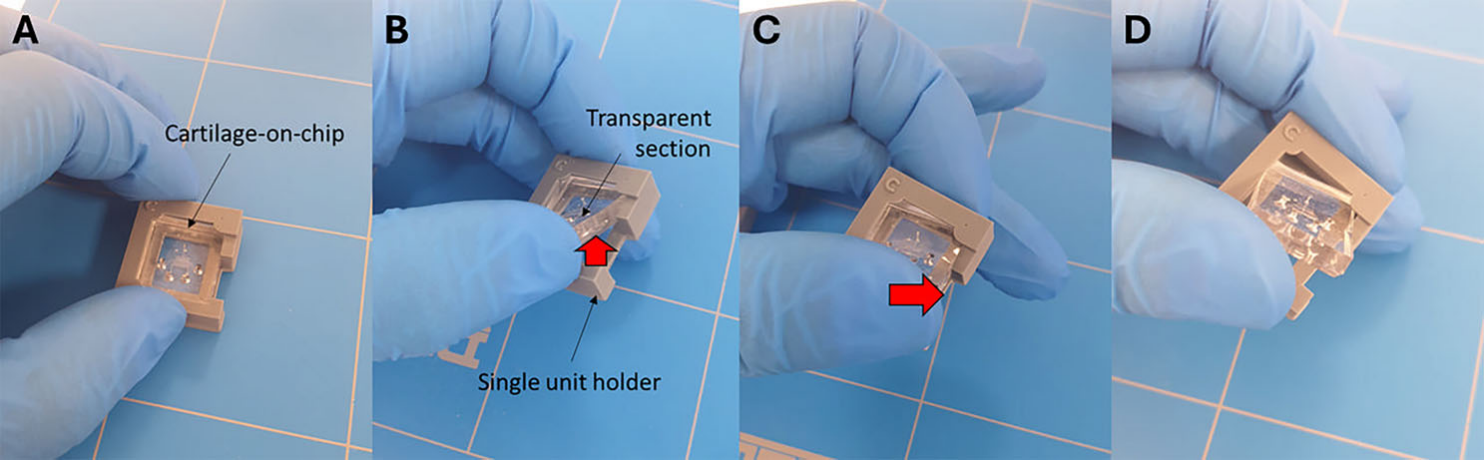
Removal of the transparent section of the cartilage‐on‐chip device from the single unit holder.

1 Clean the BSC with isopropanol or ethanol.
2 Retrieve the Cartila‐plate from the incubator.
3 Remove each unit from the Cartila‐plate to be used and place the units on the surface of the BSC.
4 Gently pull up the right‐side corner of the transparent part out of the cartilage‐on‐chip module (Fig. 14B), and gently slide it from the left to the right to remove it from the holder (Fig. 14C‐D).

**Figure 15:**
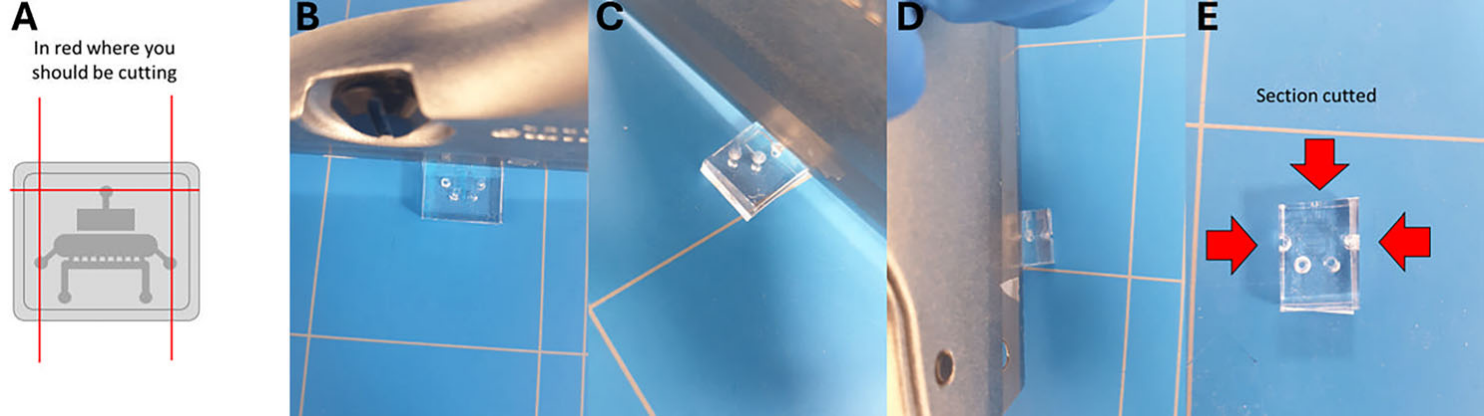
Cutting the transparent section of the cartilage‐on‐chip device with a razor blade. (**A**) An illustration of the cutting sites. (**B**‐**D**) Illustrations of the cutting of the transparent sections. (**E**) A cartilage‐on‐chip device cut from the three sides.

5 Using a sterile razor blade or scalpel cut three sides of the device as shown in Figure 15.
  Always have a sharp sterile razor blade or scalpel, as you may break the agarose inside if it is not a clean cut. The cartilage‐on‐chip device consists of a top half and a bottom half. The top half presents the actuation unit while the bottom half consists of a slab.

**Figure 16:**
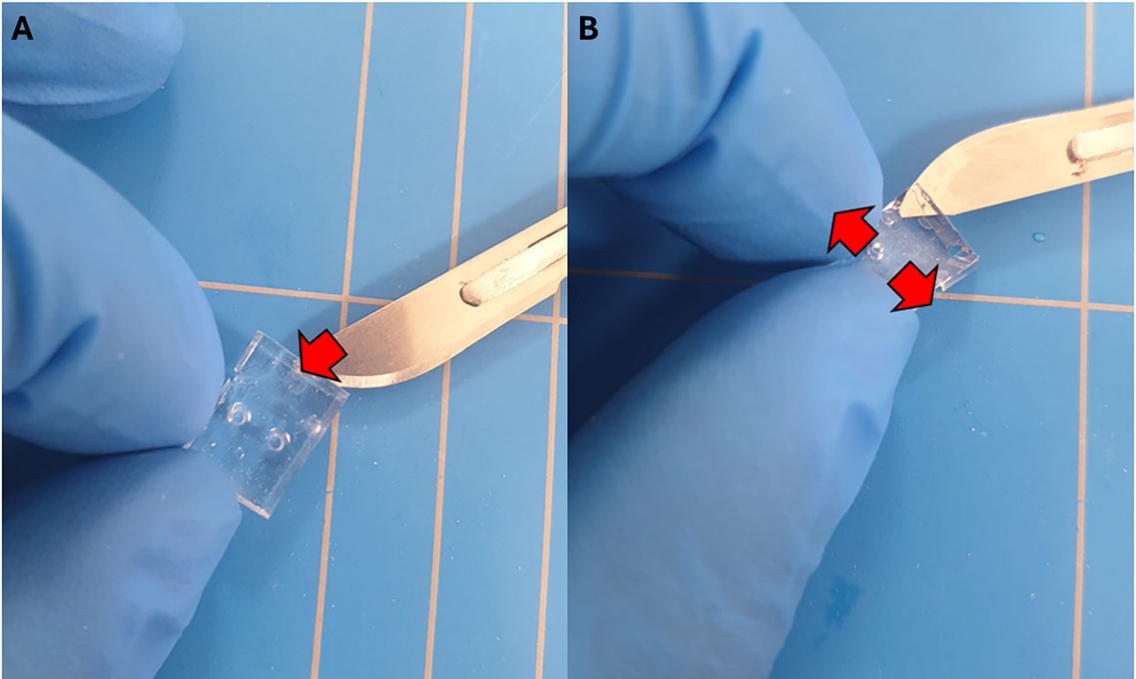
Cutting the transparent section of the cartilage‐on‐chip device's actuation channel with a scalpel. (**A**) Insertion of the blade into the actuation unit hole. (**B**) Illustration of the cut along the actuation unit.

6 Flip the device so that the bottom half is facing upwards (Fig. 16).

**Figure 17:**
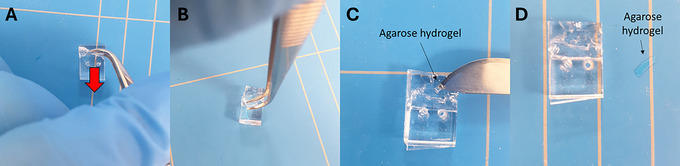
Removal of the agarose hydrogel from the transparent part of the cartilage‐on‐chip device.

7 Insert the blade into the actuation unit hole and cut along the side (Fig. 16A‐B).
8 Gently pull and remove the bottom of the device from the cartilage‐on‐chip device with the help of forceps (Fig. 17B).

9 Gently remove the agarose hydrogel from the cartilage‐on‐chip device using a razor blade or scalpel (Fig. 17C).
10 Place the agarose hydrogel on a glass slide.
11 Pipette 50 µl PBS on top of the extracted agarose hydrogel using a 200‐µl micropipette to prevent dehydration.
12 Repeat steps 4 to 11 for the required number of cartilage‐on‐chip devices.
13 Place a maximum of 2 agarose hydrogels on a glass slide.

### Agarose fixation

**Figure 18:**
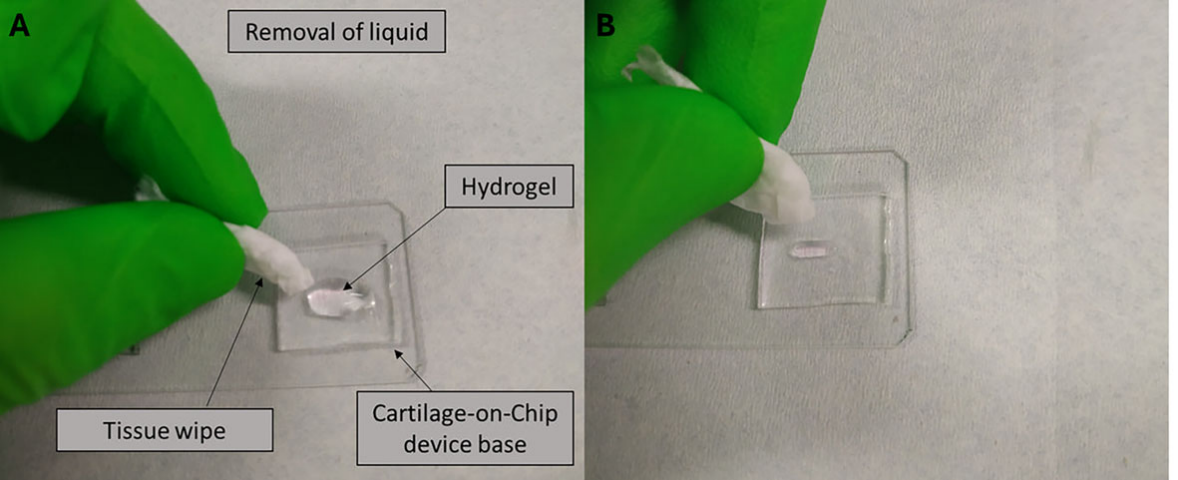
Removal of the liquid with a tissue wipe from the agarose hydrogel.

14 Remove the PBS from on top of the two agarose hydrogels using a tissue wipe (Fig. 18) or by aspirating with a 200‐µl micropipette set at 100 µl.

15 Add 50 µl of 4% formaldehyde to the agarose hydrogels using a 200‐µl micropipette.
16 Repeat steps 14 and 15 for the required number of extracted agarose hydrogels.
17 Incubate the agarose hydrogels for 35 min at room temperature with formaldehyde.
18 Remove the 4% formaldehyde using a wipe or by aspirating with a 200‐µl micropipette set at 100 µl.
19 Wash the agarose hydrogels with 50 µl PBS using a 200‐µl micropipette.
20 Remove the PBS using a wipe or by aspirating with a 200‐µl micropipette set at 100 µl.
21 Repeat steps 19 and 20 two more times.
22 Add 100 µl PBS on top of the agarose hydrogels.
23 The agarose hydrogels are now fixed.
24 Fixed agarose hydrogels can be studied using methods such as immunofluorescent (IF) labeling or immunohistochemistry (IHC).

## Basic Protocol 4 PREPARATION OF CARTILAGE‐ON‐CHIP FOR RNA EXTRACTION

This protocol outlines the procedure for preparing the chiron's cartilage‐on‐chip device for RNA extraction. RNA extraction process using an RNA kit is not described in this protocol. Depending on the number of cells inside the cartilage‐on‐chip or the type of hydrogel used, different RNA kits can be preferred. For agarose hydrogel the RNeasy micro kit (Qiagen, cat. no. 74004) is recommended. Always take the RNA dissociation buffer from the kit that will be used for the RNA extraction assay. Using the recommended kit, the expected RNA yield is 200 ng/chip from a single agarose hydrogel.

### Materials

70% (v/v) isopropanol (VWR, cat. no. 93002.1016 or equivalent)
96% (v/v) ethanol, diluted to 70% (VWR, cat. no. 20824.365 or equivalent)
Phosphate‐buffered saline (PBS) without calcium and magnesium (Thermo Fisher Scientific, cat. no. 15343571 or equivalent)
RNA dissociation buffer (i.e., Qiagen RNeasy micro kit, cat. no. 74004)

Cartila‐plate (chiron, cat. no. CoC‐0004)
Cartila‐plate lid (chiron, cat. no. CoC‐0002)
Cartilage‐on‐chip device (chiron, cat. no. CoC‐0001)
Biological safety cabinet (BSC)
20‐, 200‐, and 1000‐µl calibrated micropipettes and aerosol barrier pipette tips
1.5‐ml microcentrifuge tubes

### RNA extraction

1 Retrieve the Cartila‐plate with 6 cartilage‐on‐chip devices from the incubator and clean them with isopropanol or ethanol.
2 Remove each cartilage‐on‐chip unit from the Cartila‐plate and place them on the BSC.
3 Aspirate the medium from the top of each cartilage‐on‐chip device using a 200‐µl micropipette, and from the perfusion channels using a 20‐µl micropipette.
4 Add 30 µl PBS into each cartilage‐on‐chip into the perfusion channels using a 200‐µl micropipette. Carefully, pipette up and down 10 times to rinse the perfusion channel. Remove the PBS and discard.
5 Prepare the RNA dissociation buffer from the intended RNA kit to be used.
6 Add 50 µl RNA dissociation buffer into the perfusion channels of each cartilage‐on‐chip using a 200‐µl pipette. Vigorously pipette up and down at least 20 times. The hydrogel should be disrupted to ensure RNA from the cells is collected.
  Pressure can be applied to the cartilage‐on‐chip to ensure RNA buffer is diffused into the entire device to optimally extract the RNA from the cells.
7 Aspirate the RNA dissociation buffer and collect it into a 1.5‐ml microcentrifuge tube using a 200‐µl micropipette.
8 Repeat steps 6 and 7 two times for the same cartilage‐on‐chip device and collect the RNA dissociation buffer into the same microcentrifuge tube.
  For one cartilage‐on‐chip device, there will be one 1.5‐ml microcentrifuge tube.
9 Store the collected 1.5‐ml microcentrifuge tubes at −80°C.
10 Continue with RNA extraction according to the instructions provided by the RNA extraction kit.

## REAGENTS AND SOLUTIONS

### Ascorbic acid, 5 mg/ml

500 mg ascorbic acid (Thermo Fisher Scientific, cat. no. 105025000 or equivalent)
100 ml H_2_O, distilled
Filter sterilize with a 0.22‐µm filter
Aliquot the prepared solution into smaller volumes
Store up to 6 months at −20°C in the dark

### Complete medium

Dulbecco's modified Eagle medium (DMEM), high glucose (Thermo Fisher Scientific, cat. no. 41966052 or equivalent)
Mix in the following, up and down at least 3 times:
10% fetal bovine serum (FBS) (Thermo Fisher Scientific, cat. no. 10270106 or equivalent)
1% (v/v) penicillin‐streptomycin (Sigma‐Aldrich, cat. no. P0781 or equivalent)
0.1% (v/v) amphotericin B (Sigma‐Aldrich, cat. no. A2411 or equivalent)
0.2 mM ascorbic acid (see recipe for 5 mg/ml solution)
4 mM l‐proline (see recipe for 50 mg/ml solution)
1× non‐essential amino acids (stock solution 100×; Thermo Fisher Scientific, cat. no. 11140035 or equivalent)
Store up to 1 month at 4°C
  Before preparing medium, place the penicillin‐streptomycin, FBS, and DMEM in a 37°C water bath at until no ice is left in the tubes and bottles.
  Use isopropanol ethanol to wipe tubes/bottles and place them in the biosafety cabinet.
  It is recommended to use 0.1% (v/v) amphotericin B (250 ng/ml) when working with bovine chondrocytes.

### l‐proline, 50 mg/ml

5000 mg l‐proline (Sigma‐Aldrich, cat. no. P5607 or equivalent)
100 ml H_2_O, distilled
Filter sterilize with a 0.22‐µm filter
Aliquot the prepared solution into smaller volumes
Store up to 6 months at −20°C in the dark

## COMMENTARY

### Critical Parameters

**Figure 19:**
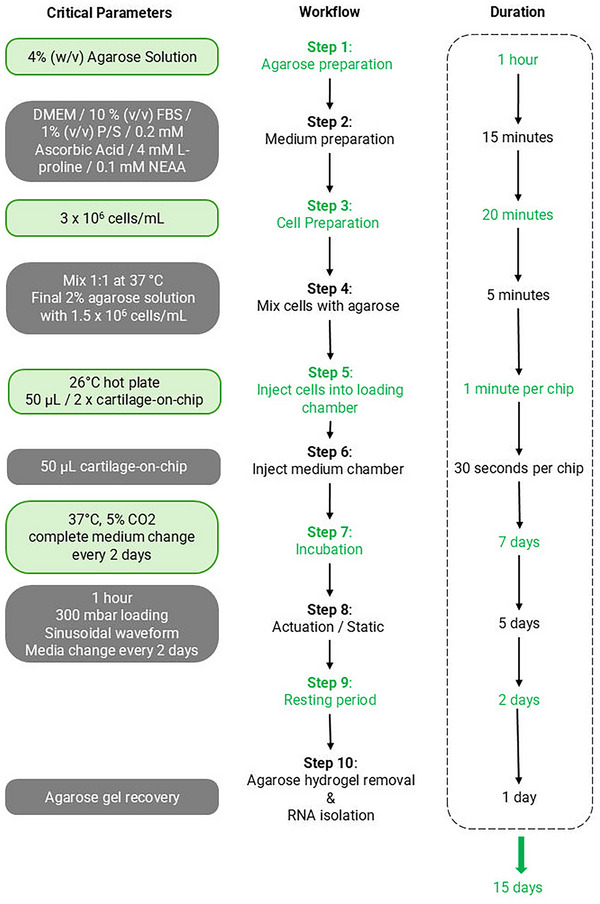
A flowchart of critical parameters, workflow, and duration. The figure was created using BioRender.

Always work in a sterile environment. Work inside a BSC and ensure all equipment is cleaned with isopropanol or ethanol before starting. Contaminations introduce variability to your experiment making outcomes unusable. Figure 19 shows all critical parameters and considerations.

#### Agarose and cell preparation

Preparing agarose often takes longer than the time allocated for it. This protocol is only meant for low melting temperature agarose. Perform a first test of your microwave as each may lead to a different melting time requirement. Optimize this step, if necessary, before using it in the models. Microwaved agarose must be kept in the water bath for at least 10 min at 57°C to guarantee uniform dissolution. Incomplete dissolution of agarose can result in uneven cell distribution and varying hydrogel rigidity within the cartilage‐on‐chip. This later compromises the mechanical properties of the gel. It is important to note that the agarose must be cooled to 37°C before mixing with chondrocytes to ensure cell viability. Chondrocytes should be washed with PBS before adding the warm trypsin, to maximize trypsin function.

The PBS‐agarose mixture should have a 4% (w/v) concentration. When the mixture is mixed 1:1 with the cell suspension (3 × 10^6^ cells/ml), a complete cell‐agarose solution results in a 2% (w/v), 1.5 × 10^6^ cells/ml final concentration. Note that the concentration of cells can vary up to 50 × 10^6^ cells/ml in the cell suspension, which is equivalent to 25 × 10^6^ cells/ml in the model. When mixing the cell suspension with the agarose solution, it is important to use an Eppendorf heating block. Lower temperatures lead to a faster agarose solution gelation, resulting in an uneven cell distribution within the hydrogel.

#### Cartilage‐on‐chip injection

Injection of the cell‐agarose suspension into the cell chamber should be done gently, otherwise, the solution can invade the perfusion channel and clog the system, making the cartilage‐on‐chip device unusable. Additionally, the complete medium should be gently injected into the perfusion channel. Excess pressure may break the agarose hydrogel.

#### Mechanical actuation

It is always recommended to perform the calibration of the pressure controller instrument before starting the experiment. This protocol uses a 300‐mbar pressure. Higher or excessive pressure can cause hydrogel damage. However, lower pressure values can lead to insufficient mechanical stimulation of the cartilage‐on‐chip. See in Figure 6 for an illustration of static, 300 mbar, and 700 mbar conditions. The mechanical actuation system in this protocol acts in a sinusoidal waveform. If this waveform is not consistently maintained, it can lead to uneven mechanical stimulation, thereby altering the experimental results.

Applying too much pressure when inserting or removing the metal wire from the actuation chamber will damage the agarose hydrogel. Always remember to carefully pipette the medium from the actuation chamber with a 20‐µl micropipette and add medium on top of the device before incubation.

#### Agarose retrieval

Agarose retrieval is a sensitive step that requires patience and special attention. Applying excessive force when handling or cutting the hydrogel can damage the hydrogel inside the cartilage‐on‐chip device.

#### RNA extraction

Remember to pipette up and down 10 times when rinsing the cells with PBS and 20 times when lysing the cells with RNA dissociation buffer. In this protocol, excessive pressure can be used when pipetting RNA dissociation buffer. It is critical to store the samples at −80°C if the RNA extraction protocol is not continued to ensure proper preservation of the RNA.

### Troubleshooting

See Table 1 for troubleshooting guidelines for the issues related to agarose‐cell suspension preparation, cartilage‐on‐chip device injection, actuation, agarose hydrogel removal, and RNA extraction.

**Table 1:** Troubleshooting Guide for Cartilage‐on‐Chip Protocols

Problem	Possible cause	Solution
Dead cells in the cell mixture	Cells are not washed before trypsinization	Wash cells before adding the trypsin
Low cell viability	Cell culture confluency is too low or high; too long trypsinization of the cells; high passage number of the cells	Primary cells are preferable, if not, use highly viable and low passage (desirable passage number is 1‐4); check cell viability before injection with trypan blue
Contamination in the cell‐agarose suspension	Usage of a non‐sterile glass beaker or other equipment during the PBS‐agarose mixture preparation	Always use sterile equipment and cover the glass beaker with tin foil, before and after the microwave
Cells in the perfusion channel	Excessive pressure during the injection of the cell‐agarose suspension into the cell chamber	Gently pipette the cell‐agarose suspension into the cell chamber
Shrinkage of the agarose hydrogel	The hot plate is not warm enough	Use a hot plate set to the appropriate temperature; let the agarose solidify for 30‐60 s before medium injection
Culture medium in the cell chamber	Excessive pressure during the injection of the culture medium	Gently pipette the cell culture medium into the perfusion channel
Uneven distribution of cells in the agarose hydrogel	Incomplete dissolution of agarose, or cell‐agarose solution not mixed properly	Make sure that the agarose is uniformly dissolved; pipette the cell‐agarose suspension up and down gently and stir the solutions using the tip of the micropipette until the two solutions are completely mixed
Air bubbles inside the cell‐agarose hydrogel	Air bubbles formed while resuspending the cell‐agarose suspension before injection	Always pipette up and down gently and keep the pipette tip inside the cell‐agarose suspension ∼2 s before taking out the tip
The agarose hydrogel ruptures after changing medium	Excessive pressure applied when aspirating the medium from the perfusion channel	Aspirate the medium with a 200‐µl pipette and then aspirate the medium inside the perfusion channel with a 20‐µl pipette; do not pipette vigorously; add the fresh medium gently
The agarose hydrogel ruptures after actuation	Excessive pressure applied when installing or removing the metal wire	Do not press the chip with your finger while installing the metal wire; gently install and remove the metal wire
	The medium was aspirated or pipetted using excessive pressure	Change the medium with the appropriate micropipette; aspirate and pipette the medium slowly so the agarose will not be disrupted
	The hydrogel dried during the actuation	Add medium on top of the perfusion channel before the actuation and check for drying during the actuation
The cells are dead or have low viability	The medium was not changed routinely, or agarose hydrogel dried during actuation	Always change the medium every 48‐72 hr; keep a small amount of medium on top of the cartilage‐on‐chip device to prevent dryness
Mechanical actuation is not happening even the pressure controller instrument shows pressure waveform	The metal wire is not plugged correctly; the plastic tubing is clogged	Check if the metal wire is inserted into the cartilage‐on‐chip devices completely; check if there is any clog inside the plastic tubing (sometimes plastic tubing ties and the airway is clogged; must change plastic tubing for a new one)

### Understanding Results

When the chondrocyte‐agarose suspension is properly injected into the cartilage‐on‐chip device at a concentration of 1.5 × 10^6^ cells/ml, it accurately simulates physiological articular cartilage. This setup mimics the walking pace of an individual when a frequency of 1 Hz and a mechanical actuation of 300 mbar is applied (Paggi, Hendriks et al., 2022). Altering the cell density within the agarose hydrogel to reflect the cell density in human cartilage, whether by increasing or decreasing the cell count, will significantly impact the results by modifying the validated parameters for a physiological cartilage‐mimicking chip. Therefore, the final concentration of 1.5 × 10^6^ cells/ml in 2% (w/v) agarose with 300 mbar pressure is anticipated to give desired results.

For example, using a higher chondrocyte concentration than the suggested 1.5 × 10^6^ cells/ml will complicate pipetting due to the increased cell density. Higher cell‐cell contact will affect the production of proteins and RNA, and greater nutrient consumption will necessitate more frequent medium changes. No comparison has been made to ensure the consistency of results between different cell concentrations.

A resting period of 1 week is suggested in Basic Protocol 1 before continuing to Basic Protocol 2. This ensures that the chondrocytes adjust to the change of environment after the stress response induced by the injection. The pro‐inflammatory screening was done to ensure this change in cytokine levels during 1 week of culture in the devices (Paggi, Hendriks et al., 2022).

### Time Considerations

Figure 1 and Figure 19 illustrate the workflow, critical parameters, and detailed time considerations for each protocol. To ensure the work is limited to weekdays, it is recommended to begin Basic Protocol 1 on a Monday. Basic Protocol 1 can be completed over seven consecutive days, from Monday to Sunday. Basic Protocol 2 can then start the following Monday and run until Friday. The weekend between these protocols is allocated for the culture of the cartilage‐on‐chip devices. On the third Monday, either Basic Protocol 3 or 4 can be initiated, depending on the experimental design. The total duration of this protocol is designed to span over 15 days, including weekends for culturing the devices.

#### Detailed time considerations for each protocol

##### Cartilage‐on‐chip injection (Basic Protocol 1)

This protocol may take up to 3 hr, depending on the number of cartilage‐on‐chip devices. The medium change step typically requires ∼30 min and is done every 48 to 72 hr.

##### Cartilage‐on‐chip actuation (Basic Protocol 2)

Preparation, actuation, and medium change together take ∼1 to ∼2 hr. It is recommended to perform the actuation at the same time each day during the actuation period to ensure consistency and produce unified results.

##### Cartilage‐on‐chip agarose hydrogel removal (Basic Protocol 3)

Depending on the number of devices, this protocol can take up to 2 hr. Fixation, which includes incubation and washing steps, generally takes 1 to 2 hr.

##### Cartilage‐on‐chip RNA extraction (Basic Protocol 4)

The duration for RNA extraction varies depending on the method chosen. However, allocate ∼1 hr for RNA dissociation from the cartilage‐on‐chip devices.

### Author Contributions

**Valtteri Peitso**: Data curation; formal analysis; investigation; methodology; validation; visualization; writing—original draft; writing—review and editing. **Zahra Sarmadian**: Data curation; formal analysis; investigation; methodology; validation; visualization; writing—review and editing. **João** Henriques: Data curation; formal analysis; investigation; methodology; validation; visualization; writing—review and editing. **Elsa Lauwers**: Data curation; formal analysis; investigation; methodology; resources; validation; visualization; writing—review and editing. **Carlo Paggi**: Conceptualization; data curation; formal analysis; funding acquisition; investigation; methodology; project administration; resources; software; supervision; validation; visualization; writing—review and editing. **Ali Mobasheri**: Conceptualization; project administration; resources; supervision; writing—review and editing.

### Conflict of Interest

Carlo Alberto Paggi and Elsa Lauwers work for chrn on‐chip biotechnology B.V. and have helped in the protocol drafting.

## Data Availability

Data sharing not applicable as no new data were generated.
